# The Training of Olympic Alpine Ski Racers

**DOI:** 10.3389/fphys.2018.01772

**Published:** 2018-12-21

**Authors:** Matthias Gilgien, Robert Reid, Christian Raschner, Matej Supej, Hans-Christer Holmberg

**Affiliations:** ^1^Department of Physical Performance, Norwegian School of Sport Sciences, Oslo, Norway; ^2^Alpine Skiing, Norwegian Ski Federation, Oslo, Norway; ^3^Department of Sport Science, Olympic Training Center, University of Innsbruck, Innsbruck, Austria; ^4^Laboratory of Biomechanics, Faculty of Sport, University of Ljubljana, Ljubljana, Slovenia; ^5^Swedish Winter Sports Research Centre, Mid Sweden University, Östersund, Sweden; ^6^School of Sport Sciences, UiT The Arctic University of Norway, Tromsø, Norway; ^7^School of Kinesiology, University of British Columbia, Vancouver, BC, Canada

**Keywords:** snow sport, elite, performance, physical demands, physical conditioning, periodization, injury, health

## Abstract

Alpine combined was the only alpine ski racing event at the first Winter Olympic Games in 1936, but since then, slalom, giant slalom, super-G, downhill, and team events have also become Olympic events. Substantial improvements in slope preparation, design of courses, equipment, and the skills of Olympic alpine skiers have all helped this sport attain its present significance. Improved snow preparation has resulted in harder surfaces and improved equipment allows a more direct interaction between the skier and snow. At the same time, courses have become more challenging, with technical disciplines requiring more pronounced patterns of loading – unloading, with greater ground reaction forces. Athletes have adapted their training to meet these new demands, but little is presently known about these adaptations. Here, we describe how Olympic athletes from four of the major alpine ski racing nations prepared for the Olympic Games in South Korea in 2018. This overview describes their typical exercise programs with respect to physical conditioning, ski training and periodization, based on interviews with the coaching staff. Alpine ski racing requires mastery of a broad spectrum of physical, technical, mental, and social skills. We describe how athletes and teams deal with the multifactorial nature of the training required. Special emphasis is placed on sport-specific aspects, such as the combination of stimuli that interfere with training, training with chronic injury, training at altitude and in cold regions, the efficiency and effectiveness of ski training and testing, logistic challenges and their effects on fatigue, including the stress of frequent traveling. Our overall goal was to present as complete a picture of the training undertaken by Olympic alpine skiers as possible and on the basis of these findings propose how training for alpine ski racing might be improved.

## Introduction

Alpine racing consists of four primary ski disciplines (events), which vary in duration, number of changes in direction, course, terrain, and jumps. The duration of a single run and the average and maximal (avg./max) speed for the primary events are as follows: slalom (SL, 52 s, 54 km/h), giant slalom (GS, 77 s, 65/85 km/h), Super-G (SG, 93 s, 86/110 km/h), and downhill (DH, 121 s, 94/150 km/h) (Reid, 2010; Gilgien et al., 2014, 2015a,b).

Alpine ski racing is a technical sport, placing high demands on the athlete’s skill and motor control (Raschner et al., 2017). Although external conditions do not change suddenly and unexpectedly, the technique involved is similar to that of open motor skill sports due to the extremely high variability in these conditions. Variations in course setting, terrain, snow conditions, speed, and visibility all place very high demands on the skier’s ability to adapt technique and tactics effectively. Furthermore, when competing, the athlete must be prepared for situations with limited knowledge of the speed at which they will approach, how their equipment will interact with the snow surface, and whether this surface has changed since the course was inspected (Reid, 2000). These considerations have important implications for how Olympic skiers should train technique in order to be prepared for competition.

In addition to technique, alpine ski racing challenges virtually all of an athlete’s physical capacities, including strength (maximal strength, strength endurance, and stability), power, aerobic and anaerobic capacity, balance, coordination and motor control, and mobility (Neumayr et al., 2003; Maffiuletti et al., 2006; Hydren et al., 2013; Polat, 2016; Gilgien et al., 2018). At the same time, these challenges are not extreme in terms of human capacity (Turnbull et al., 2009) and athletes with substantially different physical characteristics can all compete at a high level.

Surprisingly, the training required by Olympic alpine ski racers has received little attention from researchers (Hydren et al., 2013). Therefore, we asked (open interview with 10 initial questions) four members of the coaching staff (head coaches and coach/scientists with access to training records of the different training groups within the ski federations working with Olympic racers in Germany, Norway, Sweden and Switzerland to describe the nature and volume of their training with respect to ski training and physical conditioning, periodization and sport-specific challenges. Ethical considerations regarding voluntary participation of the coaching stuff, confidentiality, and anonymity were strictly followed. The information thus collected was used to arrive at generalizations concerning this training and establishing the correct order of magnitude of their training. Factors of specific relevance to Olympic ski racers are highlighted to provide insight into real-life Olympic training. Our manuscript is a first attempt to provide researchers with a holistic understanding of training, as well as the related constraints and challenges. An improved understanding has the potential to better design relevant training-related research projects related to the real-life situation of alpine ski racing. However, the accuracy of the data collected in the teams did not allow valid comparisons between women and men or elaboration on what nations should enhance in order to improve performance.

## Ski Training

Olympic alpine skiers typically train and compete for 130–150 days each year. The total volume of training and its distribution between the four disciplines depends on the skier’s specialization. Most Olympic skiers specialize either in the technical (SL and GS) or speed disciplines (SG and DH), although single-discipline specialists and athletes competing in 3 or more disciplines are not uncommon. While the majority of an athlete’s training involves his/her own discipline, Olympic skiers sometimes train other disciplines as well. For example, speed discipline specialists may train some SL to prepare for the combined event, and vice-versa. The recent addition of parallel SL events to the World Cup and Olympic Games has increased focus on this discipline. However, regardless of specialization, the total number of training days appears to be similar.

Table 1 documents typical skiing training volumes for Olympic alpine skiers. Part 1 provides examples of sessions during the preparation and competition periods for each discipline, while Parts 2 and 3 illustrate the training by an athlete who trains either two technical or two speed disciplines for an equal number of days each. The volume of competition-like training is presented both in terms of time and number of turns (excluding warm-ups, skiing to and from lifts, and the time spent on lifts).

**Table 1:** Typical volumes of ski training by Olympic alpine skiers.

	Part 1: The content of one session of ski training during the preparation and competition periods		


		*Preparation period*		*Competition period*	
	*Slalom*	6–12 runs on a partial-to-full length course × 40–60 turns		2–6 runs on a partial-to-full length course × 50–60 turns	
	*Giant slalom*	6–12 runs on a partial-to-full length course × 25–50 turns		2–5 runs on a partial-to-full length course × 25–50 turns	
	*Super-G*	4–8 runs on a partial-to-full length course × 15–40 turns		2–4 runs on a partial-to-full length course × 15–40 turns	
	*Downhill*	4–8 runs × 15–35 turns		3–6 runs × 15–35 turns	

	**Part 2: Ski training during one session/period/year in terms of time**	***Specialist technical events***		***Specialist speed events***	
	***Event***	***SL***	***GS***	***SG***	***DH***

Preparation period	*Training Days/Period*	32	35	22	25
	*Runs/Day*	8	8	6	6
	*Time/Run [s]*	40	45	50	60
	*Time/Day [min]*	5.3	6.0	5.0	6.0
	*Total Time/Event [h]*	2.8	3.5	1.8	2.5
	*Total Time/Period [h]*	6.3		4.3	
Competition period	*Days/Period*	30	32	25	32
	*Runs/Day*	4	3.5	3	3
	*Time/Run [s]*	40	45	50	60
	*Time/Day [min]*	2.7	2.6	2.5	3.0
	*Total Time/Event [h]*	1.3	1.4	1.0	1.6
	*Total Time/Period [h]*	2.7		2.6	
	*Total Time/Event [h]*	4.2	4.9	2.9	4.1
	*Total Time/Year [h]*	9.1		7.0	

	**Part 3: Ski training during one session/period/year in terms of the numbers of turns**	***Specialist technical events***		***Specialist speed events***	
	***Event***	***SL***	***GS***	***SG***	***DH***

Preparation period	*Training Days/Period*	32	35	22	25
	*Runs/Day*	8	8	6	
	*Turns/Run*	50	37	28	
	*Turns/Day*	400	296	165	
	*Total Turns/Event*	12800	10360	3630	
	*Total Turns/Period*	23160			
Competition period	*Training Days/Period*	30	32	25	32
	*Runs/Day*	4	3.5	3	3
	*Turns/Run*	55	37	28	
	*Turns/Day*	220	130	83	
	*Total Turns/Event*	6600	4144	2063	
	*Total Turns/Period*	10744			
Total Turns/Event/Year		19400	14504	5693	
Total Turns/Year		33904			


*The ranges presented are averaged range limits (rounded-off) for the nations included. Part 1: Typical sessions of ski training for each discipline during the preparation and competition periods. Parts 2 (time) and 3 (number of competition-like turns): training by an athlete who trains for either two technical or two speed disciplines. The preparation period includes all ski training from mid-March to mid-October/mid-November and the competition period both competition and training, in both cases excluding free-skiing and any skiing that does not resemble competition.*

An on-snow training session is commonly held in the morning, when the temperature is low and snow hard. Such a session begins with an off-snow warm-up and 2–5 warm-up runs of free-skiing, including technique drills, on a prepared slope. Most of the session involves partial or full-length runs on a competition-like course, with the number of runs and type of course set depending on the event and time of year (Table 1). Breaks between runs are typically 10–30 min long, depending to a large degree on the turn-around time on the lif**t**. In some cases, snowmobiles are used to reduce this turn-around time.

The length and number of runs can vary substantially, depending on the season, conditions and athlete, as shown in Table 1. Training runs are usually shorter than competition runs for two reasons. First, full-length competition runs are quite exhausting and a larger number of shorter runs is thought to provide overall higher quality training. In addition, during training teams usually do not have access to slopes that can accommodate a full-length competition course, especially in the case of the speed disciplines and GS. Other important concerns include how best to organize training to achieve optimal conditions with respect to snow, weather and slope and, during the competition period, to achieve training conditions as similar as possible as those in the next race.

Depending on the time of year, SL training consists of 2–12 runs with 40–60 turns (each lasting about 0.8 s) for a total of 100–700 changes in direction during a session. Each of these changes involves a sharp increase in ground reaction force, which can be as high as 4 times body weight (BW) (Reid, 2010; Supej and Holmberg, 2010). GS training consists of 2–12 runs with 25–50 turns (each lasting about 1.4 s) resulting in a total of 50–600 changes in direction, each involving maximal ground reaction forces of approximately 3.2 times BW (Gilgien et al., 2014, 2018).

Depending on the time of year, SG training consists of 2–8 runs with 15–40 turns (each lasting about 2.3 s) resulting in a total of 30–300 changes in direction per session. Each of these changes involves a relatively smooth increase in ground reaction force compared to GS and SL, peaking at approximately 2.6 times BW (Gilgien et al., 2014, 2018). In the case of SG about 20% of the run time is spent skiing straight, without turns (Gilgien et al., 2018).

Depending on the time of year, DH training consists of 3–8 runs, with 55% of the run time spent turning (15–35 turns). Each turn lasts approximately 2.3 s, with maximal ground reaction forces of 2.6 times BW (Gilgien et al., 2014, 2018). The remaining 45% of the run time involves skiing straight, with the skier in the tucked position on average 36.8% of the time (Gilgien et al., 2018).

## Physical Conditioning

To meet the broad physical demands of their sport, alpine skiers train strength and core stability, power, aerobic and anaerobic endurance, coordination/motor skills, balance, and mobility, together with supplementary training, often involving cross-training in other sports (Reid, 2000; Hydren et al., 2013). Strength training often targets the entire body, with special emphasis on the legs, core, and hip/gluteal region. Depending on the athlete’s individual needs, strength training can focus on strength endurance, hypertrophy, maximal strength and/or power. Compared to other sports, there is special focus on stabilization of the core and hip/pelvis region (Hydren et al., 2013), as well as eccentric training to sustain the high loads and shocks encountered when turning (Ferguson, 2010; Hydren et al., 2013; Patterson and Raschner, 2015). Training of coordination/motor control, balance and quickness involves off-snow imitation of skiing and is often combined with strength, power, or endurance training (Raschner et al., 2004; Hydren et al., 2013). The large variety of activities used for endurance training include cycling, running (on uneven terrain as well), swimming, kayaking, roller blading, and sports with intense activity such as football, hockey, and maneuvering through obstacle courses.

Within this framework, the training of Olympic skiers is adjusted to meet individual needs. The physical conditioning of specialists in technical and speed disciplines is generally similar, except that speed skiers place more emphasis on endurance and strength, while technical specialists focus on quickness and power.

During periods of physical conditioning, weekly training typically consists of 14–21 h distributed over 10–14 sessions, with a variety of training forms (Table 2). The nature of this training can vary substantially, since many Olympic skiers suffer acute or chronic injuries (Haaland et al., 2015), which require alternative training forms and continuous monitoring by health care personnel. Efficient and effective management of training is based on testing and analysis of individual fitness (Patterson et al., 2009, 2014; Hydren et al., 2013; Raschner et al., 2013). In the case of Olympic ski racers this is generally done twice annually, once at the beginning of the preparation period in May and again before the competition season in October. This assessment typically covers various aspects of strength, power, endurance and agility, although some nations have specific test protocols and tend to base their training more extensively on physiological testing than others. Moreover, at the highest level of performance individual differences are given great consideration when deciding when and how the skiers train, including the injuries that are not uncommon among elite skiers.

**Table 2:** The nature and number of weekly sessions of physical conditioning and on-snow training an athlete can choose from during the preparation period and competition week.

Preparation period; Physical conditioning: A typical week of training is composed of the training forms listed below. Altogether, skiers perform 10–14 training sessions for a total of 14–21 h
• 2–4 sessions of endurance training (aerobic and/or anaerobic, depending on the period) • 2–4 sessions of strength training • 1–2 sessions of explosive strength training/plyometrics (depending on the period) • 2–3 sessions of agility/motor training • 3–5 sessions of stability and mobility training • 1–2 sessions of cross-training in other sports (depending on the period) or team-building activities
Preparation period; On-snow training: A typical week of training is composed of the training forms listed below. Altogether, the skiers perform 10–14 training sessions for a total of 14–21 h
• 5–9 sessions of on-snow/technique training • 3–7 (daily) sessions of active recovery • 1–2 sessions of aerobic capacity (intervals) • 0–1 session of maximal/explosive lower-body strength training • 0–1 session of cross-training in other sports or team-building activities • 2–7 (daily) sessions of stability and mobility training
Competition period: normally with 1–3 competitions a week. A typical week of training and competition is composed of the training forms listed below. Altogether, the skiers train/compete 7–14 times a week
• 1–3 competitions • 1–3 official DH training runs for skiers in speed events or 1–3 ski training sessions for the other events • 4–7 sessions of active recovery • 4–7 sessions of stability and mobility training • 0–1 session of aerobic capacity (intervals) • 0–1 session of maximal/explosive lower-body strength training • 0–1 session of speed/quickness training or games • 1–3 days of travel (representing a significant load during this period)

## Periodization – Structure of the Training Year

The periodization of alpine ski racing does not adhere strictly to a traditional annual cycle based on the schedule of competition and development of the athlete’s form (Matveyev, 1981). Instead, the availability of good training conditions largely determines this periodization. The competition period lasts from October/November to March. The preparation period starts in April with on-snow ski training, followed by physical conditioning from May to July, which is then mixed with blocks of on-snow training from August to October/November. Instead of planning a single transition period following the competitive season, periods of recovery are incorporated into the program in April, May, and July.

## The Preparation Period

In April, once the tourist season has abated and public slopes become more accessible for training, skiers take advantage of the remaining natural and man-made snow to test their equipment and train basic skills. The moderate altitudes and short lift turn-around times permit a relatively high volume of training.

From the middle of May to July, the northern hemisphere is often warm, limiting the possibility for high-quality training on snow. At the same time, slope availability in the southern hemisphere is limited. Therefore, this period is typically used for sustained physical conditioning designed to achieve a lasting effect. All component**s** of conditioning are included throughout this preparation period, but initially, emphasis is placed on general physical conditioning, including strength and endurance. As summer approaches, the focus shifts to more training of maximal strength, power, and anaerobic endurance. From August to October/November, the focus is on on-snow training, interspersed with short periods of recovery and physical conditioning designed to maintain general condition and develop sport-specific qualities such as power.

The costs of traveling overseas for ski training and for preparing ski arenas (snow surface, netting/safety, etc.) require such training to be concentrated into blocks of 1–4 weeks. Teams with access to nearby glaciers have reduced travel and infrastructure costs and can choose to conduct more, but shorter (4–5 days) blocks of ski training on these glaciers. The choice between training on nearby glaciers or overseas depends not only on the cost, duration and frequency of on-snow training blocks, but also on altitude and snow conditions. To minimize accumulated fatigue and maximize training quality, the use of a larger number of shorter training blocks at moderate or low altitude is advantageous. Since the interaction between skis and snow on a glacier differs from that on natural and man-made snow and all World Cup races (except for the opening race in October) are held on the latter, it is important that equipment be tested under World Cup-like snow conditions before the season starts. This is one of the reasons why teams travel to the southern hemisphere. However, long trips, especially those over several time zones, influence the load of training and require extra recovery.

During August and September teams generally travel to train on nearby glaciers or on winter/spring snow in the southern hemisphere. During this period the athletes live at low or moderate altitude and ski at moderate or high altitude (with the exception of Chile, where skiers both live and train at high altitude).

During October, temperatures at high altitude provide good training conditions and teams prepare on glaciers in the Alps for the opening World Cup race in October. During November, or as soon as climate and snow conditions permit, training moves to ski areas with man-made snow and more variable terrain. During this period it is critical to finalize the choice and preparation of equipment, particularly for aggressive artificial snow, before the tight competition schedule begins.

## The Competition Period

Physical training during the competition period is designed to (1) maintain physical fitness; (2) achieve peak physical form for competitions; (3) engage in active recovery and rehabilitation of injuries; and (4) provide relaxation/fun (distraction from competition), and is governed by the racing schedule and on-snow training. Specifically, between races skiers prepare for technical aspects of the upcoming races (such as terrain, course setting, snow and light conditions, and adaptation of equipment), while physical fitness *per se* is not optimized to the same extent as in the case of some other sports with less varied forms of exercise, e.g., certain endurance sports.

During a typical week of competition, skiers participate in 1–3 races and perform 1–4 sessions of ski training. Physical training is adapted to travel load, recovery, and health. Technical competitions are usually conducted on the weekend, leaving weekdays for training and travel, often to a training base somewhere in central Europe. Athletes competing in DH complement their races with the official training runs permitted on the course used for competition, which greatly limits their total weekly volume of training.

A day with competition starts with a physical warm-up, followed by a skiing warm-up, inspection of the course and a second physical warm-up. For events involving several runs the physical warm-up is repeated prior to each run. During weeks without competition, training is similar to the light ski training during the preparation period (see Table 2).

When scheduling competitions, the International Ski Federation attempts to minimize the requirement for travel, especially between time zones, which is a potential risk for illness and injury (Spörri et al., 2012, 2016). Nonetheless, competition periods from November to March are continuously intense. Skiers seldom skip races in order to recover or train specifically for major events, since competition appears to be the best way to train for the snow conditions and course preparation involved in major events. Preparation of downhill courses in particular requires much time and effort, so few ski resorts allow these to be used for training during the tourist season. Accordingly, downhill races and official training associated with races offer the best training possibilities. Athletes also avoid skipping races in order to keep their starting position and enhance the potential to win prize money.

## Future Perspectives

Competition in elite sports drives continuous development of human athletic performance, always pushing limits. In the section below, we explore potential approaches to improving the training of alpine ski racers.

### Ski Training Volume

As already discussed, the total volume of competition-specific training of technique is limited by a number of physiological, psychological, and practical factors. Innovative approaches to increasing the volume of training, while optimizing recovery and health, may further improve performance. For instance, the capacity for ski training on any single day may be enhanced by elevating physical capacity through better conditioning. Further increases in ski training volume may be made possible by selecting training venues at lower altitudes, reducing the fatigue associated with exposure to high altitude. Improvements in snow-making technology and snow storage may help to counter the threat of climate change to snow packs around the globe (Pachauri and Meyer, 2014), which is steadily reducing possibilities for on-snow ski training (Wolfsperger et al., 2018). In addition, short-term weather exerts a major impact on ski training and an improved ability to adapt to unexpected changes in weather and snow conditions – for example, by limiting the size of training groups – can allow greater volume, as well as better quality. In addition, close collaboration with ski area operators may enable teams to increase training volume by prolonged access to lifts and training slopes or the use of transportation, such as snowmobiles, that reduce the turn-around time between runs. Certain modifications in the annual schedule may also allow more on-snow training, e.g., scheduling such training when it can be performed at local ski areas at low altitude. Skiing indoor is also becoming more popular, particularly during summer, when snow conditions on glaciers are deteriorate due to elevated temperatures. For many nations, on-snow training during April and May is relatively inexpensive, snow conditions are often good for long periods of the day, and training at lower altitudes is thus possible. This training is designed to maintain the athlete’s skiing skills throughout the training season and test new equipment (skis/boots) for the upcoming competition period.

### The Effectiveness of Ski Training

Regardless of how well-trained an athlete is, the volume of on-snow training will always be limited by fatigue and health issues. However, the potential to improve the effectiveness of this training in terms of learning and transferring skills to competition is essentially unlimited. For example the use and combination of holistic full course versus element training. Insights into the basic concepts of motor learning – such as the distribution, variability, and specificity of practice; the use of verbal instructions and feedback (always); appropriate employment of augmented feedback from video (following every training session), timing (always, except during the early preparation phase) and other sensors; and mental practice (Magill and Anderson, 2017) – can potentially improve ski technique training considerably. Moreover, optimizing the organization and form of ski training might enhance effectiveness. Most ski training is conducted holistically, including more or less all of the different aspects of skiing competition. To increase the number of repetitions of a certain element and improve focused learning, more element training might be beneficial. Additional focus on individual elements or certain conditions might be beneficial. For instance, climate change may lead to softer, more difficult snow conditions during competition, as well as to bumpier courses for top athletes who start late in the second run of technical events. Accordingly, **t**raining under sub-optimal snow conditions may become more important in the future.

### Physical Conditioning

The physical training of alpine skiers is complex, focusing on multiple capacities such as strength and endurance during the same period. In periods when off- and on-snow training are combined, technical training should not be compromised by fatigue due to physical training. Clearly, a better understanding of the effects of training on the various components of fitness, in combination with technical training, could guide coaches in their attempts to improve the quality and volume of physical training. More research on physical conditioning and its combination with on**-**snow training is definitely needed.

### Health, Training, and Performance

Most Olympic athletes have a history of injury and suffer from some sort of chronic injury that affects training. Thus, for many athlete’s, his/her health governs the training schedule to large extent. Balancing training loads with appropriate recovery is therefore essential for effective training, but not always easy for coaches and health care personnel to achieve in practice. The Olympic athlete might be among the athletes who have developed a good feeling for the balance between load and recovery, since this skill might be one of the reasons why they became Olympic athletes. New technology, such as wearable sensors, might facilitate finding this balance by improving quantification of the loads imposed by on-snow training and competition (Gilgien et al., 2013, 2018; Fasel et al., 2016).

## Author Contributions

MG, RR, CR, MS, and H-CH designed the study. MG, RR, and H-CH collected the data. MG analyzed the data. All authors contributed to the writing the manuscript.

## Conflict of Interest Statement

The authors declare that the research was conducted in the absence of any commercial or financial relationships that could be construed as a potential conflict of interest.

## References

[B1] Fasel B. Spörri J. Gilgien M. Boffi G. Chardonnens J. Müller E. (2016). Three-dimensional body and centre of mass kinematics in alpine ski racing using differential GNSS and inertial sensors. *Remote Sens.* 8:671 10.3390/rs8080671

[B2] Ferguson R. A. (2010). Limitations to performance during alpine skiing. *Exp. Physiol.* 95 404–410. 10.1113/expphysiol.2009.047563 19897568

[B3] Gilgien M. Crivelli P. Spörri J. Kröll J. Müller E. (2015a). Characterization of course and terrain and their effect on skier speed in world cup alpine ski racing. *PLoS One* 10:e0118009. 10.1371/journal.pone.0118119 25760039 PMC4356573

[B4] Gilgien M. Crivelli P. Spörri J. Kröll J. Müller E. (2015b). Correction: characterization of course and terrain and their effect on skier speed in World Cup alpine ski racing. *PLoS One* 10:e0118119. 10.1371/journal.pone.0128899 25760039 PMC4356573

[B5] Gilgien M. Kröll J. Spörri J. Crivelli P. Müller E. (2018). Application of dGNSS in alpine ski racing: basis for evaluating physical demands and safety. *Front. Physiol.* 9:145. 10.3389/fphys.2018.00145 29559918 PMC5845727

[B6] Gilgien M. Spörri J. Chardonnens J. Kröll J. Müller E. (2013). Determination of external forces in alpine skiing using a differential global navigation satellite system. *Sensors* 13 9821–9835. 10.3390/s130809821 23917257 PMC3812581

[B7] Gilgien M. Spörri J. Kröll J. Crivelli P. Müller E. (2014). Mechanics of turning and jumping and skier speed are associated with injury risk in men’s World Cup alpine skiing: a comparison between the competition disciplines. *Br. J. Sports Med.* 48 742–747. 10.1136/bjsports-2013-092994 24489379

[B8] Haaland B. Steenstrup S. E. Bere T. Bahr R. Nordsletten L. (2015). Injury rate and injury patterns in FIS World Cup Alpine skiing (2006–2015): have the new ski regulations made an impact? *Br. J. Sports Med.* 50 1–6. 10.1136/bjsports-2015-095467 26559877

[B9] Hydren J. R. Volek J. S. Maresh C. M. Comstock B. A. Kraemer W. J. (2013). Review of strength and conditioning for alpine ski racing. *Strength Cond. J.* 35 10–28. 10.1519/SSC.0b013e31828238be 23207888

[B10] Maffiuletti N. A. Impellizzeri F. Rampinini E. Bizzini M. Mognoni P. (2006). Is aerobic power really critical for success in alpine skiing? *Int. J. Sport. Med.* 27 166–167. 10.1055/s-2006-923854 16475064

[B11] Magill R. A. Anderson D. I. (2017). *Motor Learning and Control: Concepts and Applications.* New York, NY: McGraw-Hill Education.

[B12] Matveyev L. P. (1981). *Fundamentals of Sports Training* 1st Edn, ed. Zdornykh A. P. (Moscow: Progress Publishers).

[B13] Neumayr G. Hoertnagl H. Pfister R. Koller A. Eibl G. Raas E. (2003). Physical and physiological factors associated with success in professional alpine skiing. *Int. J. Sports Med.* 24 571–575. 10.1055/s-2003-43270 14598192

[B14] Pachauri R. K. Meyer L. A. (2014). *IPCC, 2014: Climate Change 2014: Synthesis Report. Contribution of Working Groups I, II and III to the Fifth Assessment Report of the Intergovernmental Panel on Climate Change.* Geneva: IPCC Available at: http://www.ipcc.ch/report/ar5/syr/

[B15] Patterson C. Raschner C. (2015). “Eccentric overload squats with the intelligent motion lifter: strength training for alpine ski racing,” in *Science and Skiing VI* eds Müller E. Kröll J. Lindinger S. Pfusterschmied J. Stöggl T. (Maidenhead: Meyer & Meyer Sort) 260–267.

[B16] Patterson C. Raschner C. Platzer H. P. (2009). Power variables and bilateral force differences during unloaded and loaded squat jumps in high performance alpine ski racers. *J. Strength Cond. Res.* 23 779–787. 10.1519/JSC.0b013e3181a2d7b3 19387401

[B17] Patterson C. Raschner C. Platzer H. P. (2014). The 2.5-minute loaded repeated jump test: evaluating anaerobic capacity in alpine ski racers with loaded countermovement jumps. *J. Strength Cond. Res.* 28 2611–2620. 10.1519/JSC.0000000000000436 24584044

[B18] Polat M. (2016). An examination of respiratory and metabolic demands of alpine skiing. *J. Exerc. Sci. Fit.* 14 76–81. 10.1016/j.jesf.2016.10.001 29541122 PMC5801720

[B19] Raschner C. Hildebrandt C. Mohr J. Müller L. (2017). Sex differences in balance among alpine ski racers: cross-sectional age comparisons. *Percept. Mot. Skills* 124 1134–1150. 10.1177/0031512517730730 28901201

[B20] Raschner C. Müller L. Patterson C. Platzer H. P. Ebenbichler C. Luchner R. (2013). Current performance testing trends in junior and elite Austrian alpine ski, snowboard and ski cross racers. *Sport. Orthop. Traumatol.* 29 193–202. 10.1016/j.orthtr.2013.07.016

[B21] Raschner C. Patterson C. Müller E. (2004). “Coordination and stabilization oriented strength training with multifunctional training devices in a long-term training program of young Austrian ski racers,” in *Proceedings of the 3rd International Congress on Skiing and Science*, eds Bacharach D. Seifert J. (Snowmass: St. Cloud State University), 93–94.

[B22] Reid R. (2000). *The Planning of Training for Highly Qualified Alpine Ski Racers: The Philosophies of Expert Coaches.* Available at: https://www.skiforbundet.no/contentassets/93727ceee6f845ed87a63124d4cb9ada/fagstoffalpint_planningoftraining_robertreid.pdf

[B23] Reid R. (2010). *A Kinematic and Kinetic Study of Alpine Skiing Technique in Slalom.* Available at: https://brage.bibsys.no/xmlui/bitstream/handle/11250/171325/reidphd 2010.pdf?sequence=1&isAllowed=y

[B24] Spörri J. Kröll J. Amesberger G. Blake O. Müller E. (2012). Perceived key injury risk factors in World Cup alpine ski racing an explorative qualitative study with expert stakeholders. *Br. J. Sports Med.* 46 1059–1064. 10.1136/bjsports-2012-091048 22872684 PMC3505868

[B25] Spörri J. Kröll J. Gilgien M. Müller E. (2016). How to prevent injuries in alpine ski racing: what do we know and where do we go from here? *Sports Med.* 47 599–614. 10.1007/s40279-016-0601-2 27480763 PMC5357247

[B26] Supej M. Holmberg H. C. (2010). How gate setup and turn radii influence energy dissipation in slalom ski racing. *J. Appl. Biomech.* 26 454–464. 10.1123/jab.26.4.454 21245505

[B27] Turnbull J. R. Kilding A. E. Keogh J. W. L. (2009). Physiology of alpine skiing. *Scand. J. Med. Sci. Sports* 19 146–155. 10.1111/j.1600-0838.2009.00901.x 19335589

[B28] Wolfsperger F. Rhyner H. Schneebeli M. (2018). *Pistenprparation und Pistenpflege. Das Handbuch fr den Praktiker.* Davos: WSL-Institut für Schnee- und Lawineforschung SLF. Available at: https://www.dora.lib4ri.ch/wsl/islandora/object/wsl:17135

